# Microscale Humidity Sensor Based on Iron-Coated Elaters of *Equisetum* Spores

**DOI:** 10.3390/bios14090414

**Published:** 2024-08-26

**Authors:** Yanting Liu, Zhexuan Lin, Xiaochun Li, Rui Huang, Xuewan Wu, Ruyi Deng, Kaisong Yuan

**Affiliations:** 1 Bio-Analytical Laboratory, Shantou University Medical College, Shantou 515041, China; 23ytliu1@stu.edu.cn (Y.L.); g_zxlin@stu.edu.cn (Z.L.); 22rhuang1@stu.edu.cn (R.H.); 23xwwu@stu.edu.cn (X.W.); 20rydeng@stu.edu.cn (R.D.); 2 Department of Ultrasound, First Affiliated Hospital of Shantou University Medical College, Shantou 515041, China; 23xcli@stu.edu.cn

**Keywords:** *Equisetum* spores, microscale humidity sensing, microfluidic chips

## Abstract

Humidity sensors deeply influence human manufacturing production and daily life, while researchers generally focus on developing humidity sensors with higher stability, higher linearity, rapid response time, etc. Yet, few people discuss measuring humidity in the microenvironment by miniaturizing sensor size into a microscale, in which the existing humidity sensors are difficult to reach. Accordingly, this study proposes a methodology for measuring relative humidity in the microscale by utilizing the distinctive morphologies of *Equisetum* spores across a range of relative humidities between 50% and 90%. *Equisetum* spores are responsive to changes in ambient relative humidity and remain in their original activities even after iron sputtering, which aims to endow the sensor with magnetic properties. The test performed in this study demonstrated a response time of 3.3 s and a recovery time of 3.6 s. In the first application, we employed such microscale sensors to work in the channel of the microfluidic chip or the cell migration microchip, as an example of working in the microenvironment. COMSOL Multiphysics 6.2 software was also used to simulate the change in relative humidity in such microchannels. Secondly, such microscale sensors are combined with smartphone-based microscopy to measure the humidity of the skin. These microscale sensors pave the new way to sensing humidity in microenvironments.

## 1. Introduction

As a consequence of the development of biomedicine [1,2,3], food processing [4], agricultural production [5], and other industries, the monitoring of environmental humidity is becoming increasingly important. In the biomedical field, a variety of equipment and devices, including respiratory testing apparatus [6], drug storage facilities [7], sterilizers, and cell culture incubators, necessitate the monitoring of humidity fluctuations. Additionally, humidity sensors are frequently employed in the context of food storage and food processing, with the objective of regulating production conditions. The humidity sensor is therefore a widely utilized instrument in environmental testing applications. In response to the increasing demand for humidity measurement, a diverse range of sensors have been developed [8,9,10,11]. Priya Malik et al. developed a series of SnO_2_/MCM-48 humidity sensors using SnO_2_/MCM-48 as a substrate via a hydrothermal method. The sensors demonstrated excellent stability and were employed in respiratory tests [12]. Vivek Chaturvedi et al. have developed a capacitive humidity sensor for the measurement of cutaneous hydration. The sensor utilizes a glyceryl monooleate–water system [13]. Sinha, Ankita et al. developed polymer-based textile materials for novel stretchable sensing devices. The researchers embedded moisture-sensitive polyester threads as capacitors and silver-plated polyamides as wires in disposable medical masks [14]. Wang and Luyu et al. fabricated quartz crystal microbalance humidity sensors using MgAl-LDH nanoflowers. These sensors are designed to measure the relative humidity of the environment by measuring the logarithmic frequencies occurring under different humidity conditions [15].

The use of traditional humidity sensors with rapid response, high sensitivity, and other advantages is widespread in daily manufacturing and life activities. However, these humidity sensors inevitably require an external power supply or an external detector. Additionally, the size of traditional humidity sensors can be a concern, particularly when measuring in confined spaces. Current microscale humidity sensors are normally prepared on an interdigital electrode (IDE), with the total area being at the scale of mm^2^ (such as 13 mm × 6 mm [16], 3 mm × 3 mm [17], 5 mm × 5 mm [18], etc.); such a size is unsuitable for work in microenvironments at micrometer scale such as the channels of microchips. In recent years, the development of humidity sensors has focused on the use of innovative materials [19,20,21,22], with less attention paid to the size of the area in which they are to be deployed. Only M. E. SOLOMON’s work in 1957 has addressed the specific issue of measuring humidity in narrow spaces. He proposed the use of cobalt thiocyanate paper to measure relative humidity in a variety of environments by matching the color of the paper to the humidity value. This method could potentially be used to assess atmospheric humidity in narrow spaces or crevices, such as floor crevices, under bark, within tree trunks, or in small tubes [23]. Despite the ongoing miniaturization of humidity sensors [24,25,26,27], there has been no report on the development of sensors capable of measuring humidity in a space at the micron level. When measuring humidity levels in small spaces, it is often the case that the dimensions of existing sensors preclude their use.

Herein, we developed a humidity measurement device capable of measuring the humidity of tiny spatial environments, applied in a 500 μm-wide microfluidic chip, which is capable of detecting the change in its relative humidity in real time. It shows great potential to meet the demand for sensing humidity in a microenvironment. The microscale sensor is fabricated by sputtering the elaters of *Equisetum* spores with iron layers, in which the *Equisetum* spores were able to respond to the changes in relative humidity, thus showing different morphologies under different relative humidity conditions, curling up at higher relative humidity and stretching out at lower relative humidity [28]. In addition to exhibiting magnetic properties following the sputtering of iron, the spores are capable of being immobilized within the microfluidic channel and retaining their responsiveness to alterations to the relative humidity. Furthermore, a camera equipped with a mobile phone microscope was applied to make it easy to observe spores change under different relative humidities. This led to the development of a portable device for measuring changes in the relative humidity of skin. The utilization of *Equisetum* spores as humidity sensors has the advantages of being non-toxic, environmentally friendly [29], low cost, portable, and able to measure the relative humidity changes in small spaces in different fields.

## 2. Materials and Methods

### 2.1. Materials and Instrumentation

The *Equisetum* spores used in this work were collected from the local outdoors; they were dried and stored in a sealed bottle for the following experiments. Iron sputtering target materials were purchased from Nanchang City, China, Hanchen New Material Technology Co., Ltd.

A coaxial illumination microscope (Shenzhen City, China, Sanchan Taida Optical Instrument Co.), KT-Z1650PVD-compact magnetron sputtering instrument (Zhengzhou City, China Keyan Instrument Co., Ltd.), LF50-fluorescence inverted microscope (Guangzhou City, China, Laite Optoelectronic Technology Co., Ltd.), biological microscope (Tokyo, Japan, OLYMPUS), Puzhong 51 microcontroller (Shenzhen City, China, Puzhong Technology Co., Ltd.), DHT22 temperature and humidity sensor (Shenzhen City, China, XuanTeJia Electronics Co., Ltd.), ZEISS Sigma 300 scanning electron microscope (Oberkochen, Germany) coupled with an X-ray spectrometer (Carl Zeiss AG), LSP02-1B-syringe pump (Baoding City, China, Longer Precision Pump Co., Ltd.), mobile phone microscope (Wuhan City, China, Convergence Technology Co., Ltd.), and Bambu A1 mini 3D printer (Shenzhen City, China, Bambu Lab Co.) were used in this study.

### 2.2. Morphological Changes in Spores at Different Relative Humidities

Using a 3D printer, we printed a rectangular space with a total volume of approximately 10 cm^3^, leaving the lower bottom empty to stick on a slide, thus allowing the observation of spore morphology under a microscope. A DHT22 temperature and humidity sensor was placed on the left side to measure the relative humidity around the spores; the measurement accuracy of the sensor was ±2% RH and the resolution was 0.1% RH [30]. The top was then covered with a coverslip to maintain an airtight environment. At the bottom of this space, a 0.2 × 2 × 0.55 cm rectangle separated the bottom into two sides, one of which held the spores and the other hot water to create a high-humidity environment (shown in Figure S1). Spores were placed on the bottom side, and the device was placed under a biomicroscope for observation, recording the current relative humidity of the air with the morphology of the spores. The next step was to add hot water to the other test at the bottom and cover the top with a slide to maintain an airtight environment. At this point, the relative humidity in the confined space rose more rapidly and the spores immediately curled up. When the relative humidity was no longer increasing and the spore morphology was no longer changing, the morphology of the spores was recorded. As the observation period was extended, a gradual decline in the temperature of the hot water within the confined space was noted, accompanied by a corresponding reduction in the relative humidity on the spore side. Additionally, the spores exhibited a gradual extension from their curled state. Finally, the upper slide was moved away slightly to allow the relative humidity to drop to the same humidity as it started at. The extent of the distance between the corresponding neighboring elaters under different relative humidity conditions was measured using ImageJ 1.54g software.

### 2.3. Response/Recovery Time of Equisetum Spores 

The iron-coated Equisetum spores were observed under a coaxial illumination microscope. After five minutes of wearing gloves, the humidity of the finger skin increases. At this point, the gloves were removed and the fingers were brought into proximity with the spores. The spores were observed under a microscope. It is possible to discern a change in morphology. The time at which the spores began to stretch and curl was recorded. Subsequently, the fingers were removed from the spore, and the time and morphology of the spore from curling to stretching at this point were documented. The relative humidity of the environment was also quantified with a DHT22 temperature and humidity sensor, and the recorded humidity values were the initial relative humidity values of the spores. After a five-minute interval, the gloves were removed, and the relative humidity was measured. This was carried out to ascertain the relative humidity of the fingers when in proximity to the spores. Measurements can be taken five times in parallel, and the average can be calculated. The final calculation yields the sensitivity, S [31]. The expression for S is as follows:

$$S=\frac{E_{x}-E_{0}}{{RH}_{y}-{RH}_{0}}$$

where $E_{x}$ is the extent of the spore as it stretches, $E_{0}$ is the extent of the spore as it curls up, ${RH}_{y}$ is high relative humidity, and ${RH}_{0}$ is low relative humidity.

### 2.4. Fabrication of Iron-Coated Equisetum Spores 

A 3D printer was applied to print a square border of 2 cm × 2 cm × 0.4 cm with a thickness of 0.2 cm, which was glued to a coverslip to form a square slot. Spores were sprinkled onto a coverslip by tapping the *Equisetum* sporangium spike on the top; the square border served to avoid air currents blowing the spores away when sputtering. Finally, it was fixed on the carousel of the magnetron sputtering apparatus. Following pre-sputtering at 200 mA for 60 s, the iron was then deposited at 300 mA for 200 s; the speed of the motor was 5. The spores turned black after the sputtering was completed.

### 2.5. Characterization of the Microscale Humidity Sensors

Magnetic characterization: A piece of white paper was cut and glued under the slide, which was then placed on a magnet. The spore should be positioned on the slide and observed under a coaxial illumination microscope. 

Scanning electron microscopy (SEM) and energy-dispersive X-ray spectroscopy (EDS): A limited number of spores were affixed with 1 cm × 1 cm conductive adhesive, and their surfaces were coated with gold before the test. The microscopic morphology of spores without iron sputtering and after iron sputtering was observed using scanning electron microscopy at a test voltage of 3 kV. EDS was employed to analyze the elemental distribution of Fe of the two spores at a test voltage of 10 kV.

### 2.6. Humidity Changes in Spores in Microfluidic Chip Channels

Spores were delivered into the microfluidic chip channel using a microfluidic flat tip and placed under a biomicroscope to observe the position as well as morphology of the spores, at which time the DHT22 temperature and humidity sensor showed a relative humidity of 50.9%. A 20 mL syringe was filled with water and then the water was expelled, after which air was introduced, resulting in the formation of wet air within the syringe. A syringe pump was used to push the wet air into the channel at a speed of 0.11 mm/s, then measurements were taken three times, and the relative humidity displayed by the DHT22 temperature and humidity sensor was 54.6%, 54.6% and 54.5%, respectively. The average value was calculated and represented; the relative humidity of the wet air at this time was 54.6%. The morphology of spores was observed in the microfluidic chip channel before the syringe pump was turned on until it was paused (Figure S2).

### 2.7. Fabrication of Smartphone-Based Device for Skin Humidity Measurement

The lab supplies needed for this setup are double-sided adhesive, an *Equisetum* sporangium spike, scissors, cling film, a filter membrane, a mobile phone microscope, and a mobile phone. The first step is to take a piece of the filter membrane, put double-sided adhesive on the filter membrane, and form a quadrilateral with a space in the middle. Gently tapping the *Equisetum* sporangium spike causes the spores to scatter on the filter membrane, which should then be covered with cling film, with the excess cut off. The next step is to stick the mobile phone microscope onto the phone camera. The final step is to apply double-sided adhesive to the filter membrane and attach it to the lens of the mobile phone microscope, taking care to lay it flat at this point so that it does not focus.

## 3. Results and Discussion

### 3.1. Microscale Humidity Sensing Strategy Based on Iron-Coated Elaters of Equisetum Spores

**Figure 1:**
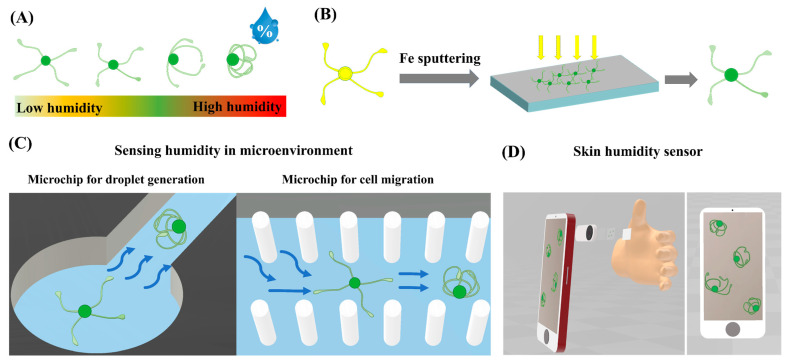
Iron-coated spores as the new humidity sensors to work in the microenvironment. (**A**) Schematic illustration showing elaters’ extent as a function of the relative humidity; (**B**) schematic illustration showing the process in fabricating iron-coated spores by magnetron sputtering; (**C**) schematic illustration showing the iron-coated spores to sense humidity in a microenvironment such as the chamber or channel of the microchip (for droplet generation or cell migration) or (**D**) combined with the smartphone-based microscopy to sense the humidity of the skin.

Figure 1 illustrates the concept of our strategy in developing microscale humidity using iron-sputtered *Equisetum* spores. As shown in the schematic illustration in Figure 1A, the four elaters of the *Equisetum* spore were unfurled alongside a decrease in humidity. The ability of the spore’s elaters to assume different shapes in response to the changes in humidity is attributed to their bilayer structure [32]. The inner layer, which is composed of cellulose, is tightly packed and longitudinally oriented, exhibiting a high density. The outer layer, on the other hand, consists of non-cellulosic polysaccharides, which have a lower density. Consequently, when the humidity of the surrounding environment fluctuates, the outer layer of the elaters becomes larger due to its lower density and greater capacity to absorb water molecules, thereby more effectively reflecting the sensitivity to humidity. The inner and outer layers of the elaters exhibit distinct characteristics due to the size of the volume change. The outer layer has a larger volume change, which causes the elaters to bend towards the inner layer when they sense a change in humidity [33]. To enable the humidity sensor with magnetic guided behavior or avoid the influence of outside airflow, the humidity sensors were prepared by sputtering *Equisetum* spores with an iron layer, as illustrated in Figure 1B. Different from other humidity sensitive sensors, such tiny spores are at the microscale in size, giving them great potential to go inside some confined space or microenvironment, such as the chamber or channel in the microfluidic chip to sense humidity (Figure 1C). This would show greater significance in some special microfluidic models, such as mimicking the human airway or studying the cells growing on an air–liquid interface [34,35,36]. Apart from that, such microscale humidity sensors were also combined with homemade smartphone-based microscopy for skin humidity sensing (Figure 1D).

### 3.2. Humidity-Responsive Properties of the Microscale Sensor

**Figure 2:**
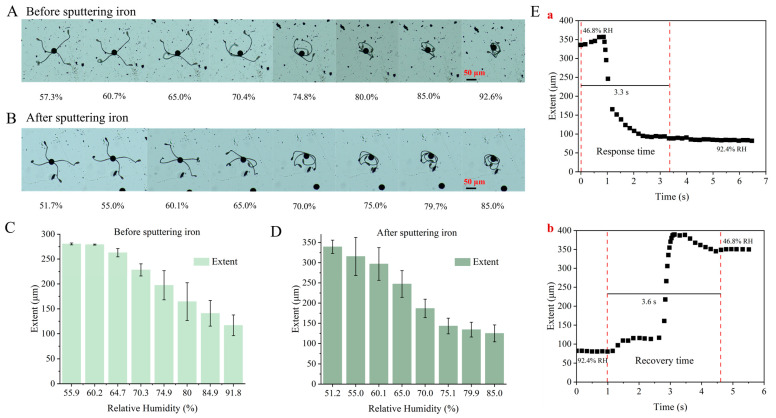
Humidity-responsive properties of the microscale sensor, different morphology of the elaters of *Equisetum* spores before (**A**) and after (**B**) iron sputtering under different humidity conditions, and corresponding extent diameter of the spores before (**C**) and after (**D**) iron sputtering under different humidity levels. Each data point was obtained from three repetitive measurements using the same humidity microsensor. (**E**) Response time (**a**) and recovery time (**b**) of the iron-coated spores.

To further characterize humidity-responsive properties of the as-proposed microscale sensor, different humidity conditions were set to observe the morphology of the elaters. As shown in Figure 2A, the elaters of *Equisetum* spores exhibit different shapes under different humidity conditions, which can reflect the real-time humidity changes for the outside environment (please note that these images are taken from Figure S3C, sensor 3 and Figure S3D, sensor 6). When the relative humidity (RH) is between 50% RH and 60% RH, the spore elaters will be farther away from each other and will spread out. As humidity increases, the elaters of the spore gradually begin to bend and form an arc shape when the RH reaches above 60%. When the RH reaches a value of 80% or above, the elaters exhibit a process of curling into a ball, thereby enclosing the spores in a protective sheath. Figure 2C also shows the corresponding relationship between humidity and elater diameter, in which the diameter decreases along with the humidity increases. In sum, in environments with low humidity, the elaters are in a stretched state, with a longer distance between neighboring elaters. Conversely, when the RH reaches above 85%, the elaters curl around the spore, and the distance between neighboring elaters is shorter (less than 150 μm).

Our main goal here is to pursue the development of a microscale humidity sensor to be able to work in the microenvironment or some specific confined space. From Figure 1, we can see that such *Equisetum* spores are at the size of a microscale, representing an excellent candidate to meet such demand. Therefore, we could put it into microfluidic chips to directly measure humidity in such a tiny chamber or channel, which is impossible to realize by other existing humidity sensors. However, since the measurement of humidity in the microfluidic chip channel requires the input of humid air at a certain velocity into the channel, and due to the fact that spores are light and easily blown away by the airflow, iron coating is sputtered onto the spores by magnetron sputtering apparatus, and then magnets are applied to hold the spores in place to avoid them being blown away by the airflow. From Figure 2B,D, we can conclude that the sputtering of iron coating on *Equisetum* spores shows no apparent influence on the property of the spores in response to humidity. We also choose different microsensors before and after iron sputtering to see the variations and, as shown in Figure S3, for different microsensors, they all show a very similar humidity response trend.

In addition, we tested the long-term stability and heat resistance of the spores. To assess the long-term stability of the humidity sensors, one batch of spores was sputter-coated with iron, and their response to humidity changes was measured both within one week and after 57 days. Figure S7 illustrates that the humidity sensor retains its optimal humidity response even after an extended period of storage (please note that for the sample “sputtering within 1 week”, we measure it using the same spore as in Figure S3D, sensor 5). Spores were placed in a high-temperature environment at 100 °C for 30 min and then experimentally tested for the relative humidity response. The findings indicated that the spores retained a good response to variations in relative humidity (Figure S8).

Figure 2E is a plot of the length of spores in response to changes in relative humidity versus response time. Figure 2(Ea) illustrates the immediate alteration in relative humidity in the vicinity of the spore when the finger is near the spore. The spore is initially observed to be fully stretched and then undergoes a rapid curling motion. At t = 3.3 s, the spores had fully curled up, indicating that the response time of the spores was 3.3 s when the relative humidity was altered from 46.8% to 92.4%. The sensitivity, designated as S, was calculated to be 5.4 μm/%RH. Figure 2(Eb) illustrates that following the displacement of the finger from the spore, at t = 1.0 s, the relative humidity of the surrounding environment begins to decline, concurrently with the gradual stretching of the spore. When t = 4.6 s, the spores were fully stretched, indicating a response time of 3.6 s for the spores when the relative humidity was changed from 92.4% to 46.8%. The results showed that *Equisetum* spores were characterized by a comparatively excellent response/recovery time (3.3 s/3.6 s). The response time for changing from 46.8% relative humidity to 92.4% relative humidity is only 3.3 s, and the response time for changing from 92.4% relative humidity to 46.8% relative humidity is only 3.6 s. Compared to the sensitivity and response/recovery time of existing sensors (please refer to Tables S1 and S2), our humidity sensor shows excellent responsiveness.

### 3.3. Characterization of the Microscale Humidity Sensor

**Figure 3:**
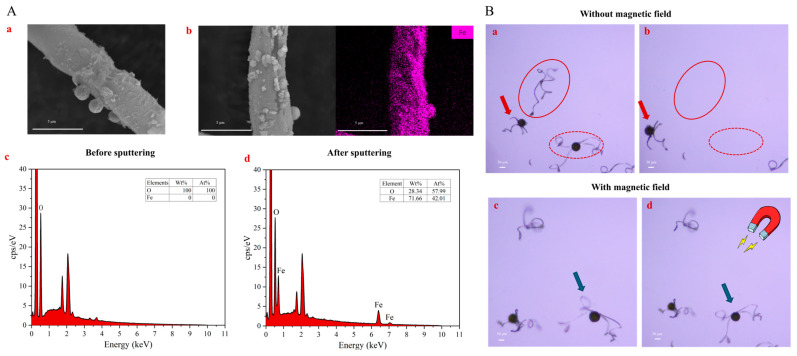
Characterization of the microscale humidity sensor, (**A**) SEM of the elater before sputtering (**a**), and EDS point scanning result (**c**); SEM of the elater after sputtering and corresponding EDS mapping of Fe element distribution (**b**); and EDS point scanning result (**d**). (**B**) Microscopy images showing magnetic properties of the iron-coated spores, Without magnetic field, before (**a**) and after (**b**) airflow passage; With magnetic field, before (**c**) and after (**d**) airflow passage.

Next, we employed scanning electron microscopy (SEM) coupled with energy-dispersive X-ray spectroscopy (EDS) to prove whether the iron had been successfully sputtered on the spores. As illustrated in Figure 3A, the morphologies of elaters show no apparent differences before and after sputtering. This is due to the fact that the sputtering layer is normally very thin at several nanometer scales, which cannot be differentiated by the SEM. However, such a thin iron layer is important for the spores to retain their original ability to sense humidity, on the premise of a desirable magnetic response. Corresponding EDS point scanning results also show that the relative content of iron was 71.66% in the sputtered spores (only O and Fe elements are taken into account here) and almost 0% in the non-sputtered spores. Therefore, we conclude that iron was successfully sputtered onto the spores with an even distribution. 

To further evaluate the magnetic performance of the iron-coated humidity sensor, the sputtered spores were subjected to airflow with and without an external magnetic field, and their motion behavior was observed under the microscope. As shown in Figure 3(Ba,Bb), without the magnetic field, the spores move to another position (indicated with a red arrow) or even move away outside the field of the microscopy (indicated with a red oval). With the application of a magnetic field (Figure 3(Bc,Bd)), the spores are confined in their original positions only with slight rotation (as indicated by the blue arrow). Such results indicate the effectiveness of magnetic sputtering, which prevents them from being blown away by the airflow in the microfluidic channel.

### 3.4. Sensing Humidity in the Microenvironment and Related Numerical Simulation

**Figure 4:**
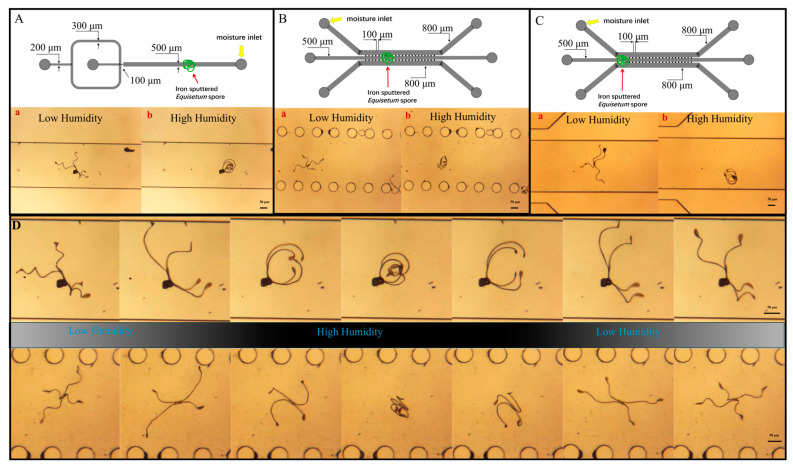
Microscale humidity sensors to work in the channel of the microfluidic chip. (**A**) Schematic illustration of the inner structure of the cross-shaped microfluidic chip and real image of the elaters of the *Equisetum* spores sensing the humidity in the microchip channel, in low (**a**) and high (**b**) humidity; (**B**,**C**) show the cell migration microchip with the microsensors in different positions (as indicated in red arrows), in low (**a**) and high (**b**) humidity. (**D**) The elaters of the *Equisetum* spores in the microchip channel to sense different humidity levels in the cross-shaped microfluidic chip (upper) and the cell migration microchip (down).

To access the humidity sensing ability of our microscale sensors in the microenvironment, two distinct microfluidic chips were utilized: a cross-shaped chip (for droplet generation) and a cell migration chip. By observing elater changes in *Equisetum* spores, we could realize measuring the relative humidity under the microenvironment in the microfluidic chip channel, a capability that is not afforded by other humidity sensors. At this juncture, the spores were fed into the microfluidic chip channel. At the time, the relative humidity of the channel was relatively low, and the spores’ elaters were in a stretched state (Figure 4(Aa,Ba,Ca)). To test if the air flow will influence on humidity sensing, the initial introduction of dry air at a velocity of 1 mm/s was undertaken. The findings indicate that the presence of dry air flow does not result in any discernible stretching or curling of spores (Figure S4, please note that pictures are taken from the measurement described in Figure 4A, the time when dry air was injected). Subsequently, by passing air with a certain humidity, the spore’s elaters sensed that the relative humidity in the channel became higher, the elaters becoming curled (Figure 4(Ab,Bb,Cb)). As illustrated in Figure 4B,C, the spores demonstrated the capacity to retain their intrinsic characteristics, namely the ability to elongate at low humidity and curl up at high humidity, across a range of locations on the microfluidic chip. In order to inject the wet air into the channel, it is necessary to utilize the syringe pump to inject the air at a specific speed and rate, during which time the relative humidity in the microfluidic chip channel reaches a peak. After the syringe pump is paused, the relative humidity in the channel declines at a gradual pace. As illustrated in Figure 4D, in the cross-shaped and cell-migrating microfluidic chip channels, spores were gradually curled by the elaters due to the elevated relative humidity and then slowly stretched as the relative humidity decreased. This reversible change in shape enabled repeated measurements of relative humidity changes in the channels. Furthermore, in microfluidic channels, there may be some spaces smaller than 400 μm where spores may be constrained while in low humidity. However, spores can utilize the curling and stretching of elaters to leave the obstacle (Figure S6). In some spaces smaller than 100 µm, fragmented segments of spores can be utilized to observe changes in relative humidity (Figure S5). 

Although the simulation of proposed experiments cannot replace real experiments, it helps to better understand physical processes or phenomena and to visualize experimental phenomena. In order to simulate the laminar flow process of a compressible fluid through a channel, COMSOL Multiphysics software 6.2 was employed. When modeling, it is essential to select 3D space and select the material type as PDMS material, after establishing the microfluidic chip model. Additionally, the fluid in the channel should be set as air. In the physical field section, the laminar flow and moisture transport in the air must be added. Furthermore, the initial relative humidity of the microfluidic chip channel and the velocity of the air input and relative humidity must be set. The Physical Field Laminar Flow Interface is employed for the calculation of velocity and pressure fields pertaining to single-phase fluid flow in a laminar state. Navier–Stokes equations are employed to elucidate the fluctuations in fluid pressure within a microfluidic channel [37].

$$\rho \frac{\partial u}{\partial t}+\rho \left(u\cdot \nabla \right)u=\nabla \cdot \left[-pI+K\right]+F$$

$$K=\mu \left(\nabla u+{\left(\nabla u\right)}^{T}\right)-\frac{2}{3}\mu \left(\nabla \cdot u\right)I$$

It can be reasonably deduced that:

$$\rho \frac{\partial u}{\partial t}+\rho \left(u\cdot \nabla \right)u=-\nabla p+\nabla K+F$$

where u is the fluid velocity, p is the fluid pressure, ρ is the fluid density, μ is the dynamic viscosity of the fluid, I is the unit matrix, T is the temperature, and F is the external force applied to the fluid.

These equations are always solved together with the continuity equation:

$$\frac{\partial \rho }{\partial t}+\nabla \left(\rho u\right)=0$$

**Figure 5:**
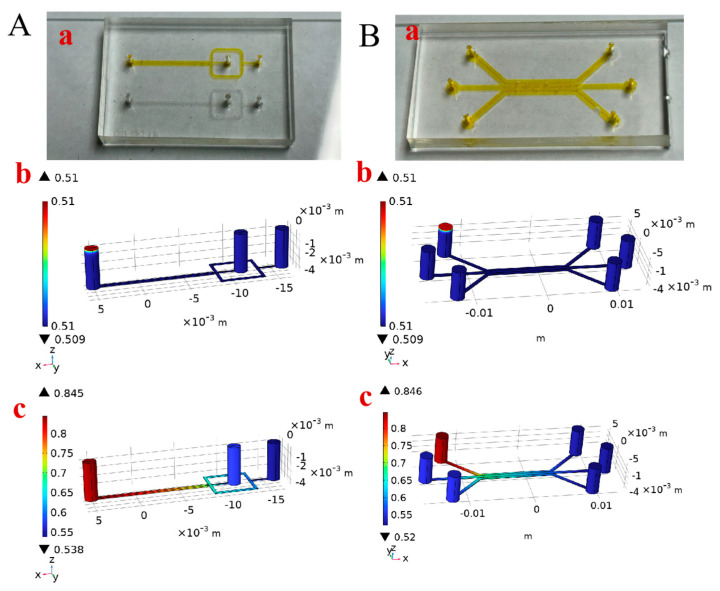
(**A**) Real image of the cross-shaped microfluidic chip (**a**) and corresponding simulation mapping of the relative humidity variation (**b**,**c**); (**B**) real image of the cell migration microchip (**a**) and corresponding simulation mapping of the relative humidity variation (**b**,**c**).

The simulation of moisture transfer by vapor diffusion and convection in humid air was conducted. The moisture content variation is expressed through the transport of vapor concentration.

$$M_{V}\frac{{\partial c}_{V}}{\partial t}+M_{V}u\cdot \nabla c_{V}+\nabla \cdot g=G$$

$$g=-M_{V}D\nabla c_{V}$$

$$c_{V}=\emptyset c_{sat}$$

where $M_{V}$ is the molar mass of water vapor, $c_{V}$ is the vapor concentration, u is the fluid velocity, g is the gravitational acceleration, ∅ is the relative humidity, $c_{sat}$ is the saturation concentration of vapor, D is the vapor diffusion coefficient of air, and G is the moisture source. Figure 5(Aa,Ba) show the physical drawings of the cross-shaped microfluidic chip (for droplet generation) and the cell migration microfluidic chip. Two different microfluidic chip models were built using COMSOL Multiphysics; Figure 5(Ab,Ac,Bb,Bc) show the simulation mapping of the relative humidity variation in the two microfluidic chip channels. Model validation and parameter analysis were performed in accordance with the experimental conditions by setting the initial relative humidity of the microfluidic chip channel at 50.9%, the air entry velocity at 0.11 mm/s and 54.6% relative humidity and simulating the change in relative humidity in the channel. As shown in Figure 5(Ab,Bb), at t = 0 s, humid air has not yet entered the channel and the relative humidity in the channel is 50.9%. As illustrated in Figure 5(Ac,Bc), at t = 25 s, the relative humidity in the channel in proximity to the inlet increases at a faster rate when air with a relative humidity of 54.6% enters the channel. In general, the relative humidity in the channel rises gradually, and there is a gradient change in the relative humidity. In the study, the gases are assumed to be ideal gases, which are carried out under transient conditions. The findings indicated that the relative humidity increased with time when the syringe pump was operational. Subsequently, the magnitude of the relative humidity in the channel after a specific interval was evaluated in conjunction with the morphology of the spores in the experimental phenomenon, thereby substantiating the accuracy of the model. It is thus feasible to approximate the relative humidity at the present moment based on the morphology of the spores within the channel.

### 3.5. Microscale Sensor Combined with Smartphone-Based Microscopy for Skin Humidity Measurement 

In another application, a microscale humidity sensor was employed for skin humidity sensing. Since the spores are unable to be observed by the naked eye, a portable device for measuring the relative humidity in the environment has been created by combining a mobile phone and a mobile microscope. Such a device comes from a recently published study from our group, in which smartphone-based polarized light microscopy was developed for on-site pharmaceutical crystallinity characterization [38]; here, we simply need to remove the two polarizers. The device has the ability to detect the change in relative humidity on the skin. This feature has been derived from the ability of *Equisetum* spores to perceive changes in relative humidity.

**Figure 6:**
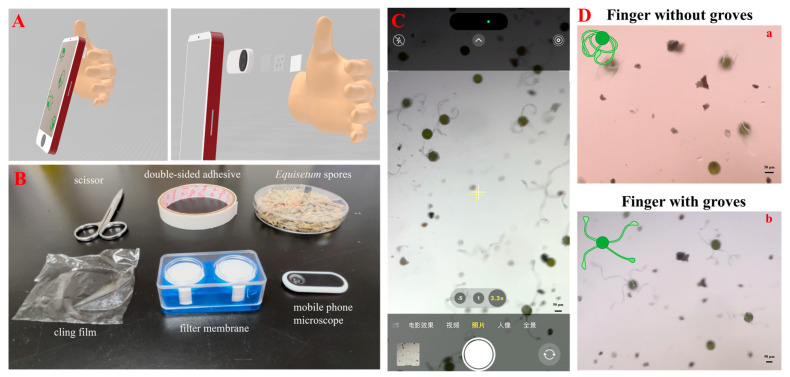
(**A**) Schematic illustration showing the strategy of using smartphone-based microscopy combined with spores for skin humidity sensing; (**B**) the elements that were used for fabricating the portable skin humidity sensing device, except for the smartphone; (**C**) a screenshot of the smartphone while sensing skin humidity using portable microscopy (Under the smartphone’s photo function interface); (**D**) use of the as-proposed portable humidity sensing device to sense finger humidity without (**a**) and with (**b**) gloves.

Figure 6A presents a schematic illustration showing the integration of the *Equisetum* spores to smartphone-based microscopy for skin humidity sensing, in which spores are positioned between the cling film and the filter membrane and subsequently affixed to the mobile phone microscope. The filter membrane serves to transfer the humidity from the external environment to the spores, while the shape of the spores is observed through the camera of the mobile phone so that the relative humidity can be measured. Figure 6B shows all the elements (except for the smartphone) that were used to fabricate such a device. Figure 6C shows a screenshot of the smartphone during the humidity sensing process using our proposed strategy. Figure 6(Da) illustrates the response of spores to an increase in relative humidity as the finger (without groves) approaches the filter membrane. At this time, the humidity is different between the inside and outside of the filter membrane, and the humidity is transferred from the outside of the device to the inside of the device. The humidity of the finger is transmitted around the spores via the filter membrane, and the spores respond to the change in relative humidity by curling up due to the increased humidity. Figure 6(Db) illustrates the shape of spores with a gloved finger in close proximity to the filter membrane. Because the gloves isolated the fingers from humidity, the relative humidity inside and outside of the filter membrane did not change, so the spores remained in their stretched form. The experimental results show that a portable device for the measurement of relative humidity has been successfully constructed using *Equisetum* spores, which can be used in real life to detect changes in the relative humidity of the skin. A video has been recorded to show the working process using our portable device for skin humidity sensing (Video S1).

## 4. Conclusions

Herein, we proposed a novel microscale humidity sensor for working in confined spaces (microenvironments) combined with smartphone-based microscopy for skin humidity measurement. It was demonstrated that relative humidity within a small space may be ascertained by means of a morphological analysis of spores. In order to prevent the displacement of spores within the channel as a consequence of airflow, spores were subjected to sputtering with iron, and the successful deposition of iron onto the spores was demonstrated through SEM and EDS. Experimental evidence has demonstrated that spores exhibit magnetic properties, curling up in environments with higher humidity and stretching out in those with lower humidity. Furthermore, they retain the capacity to respond within a relative humidity range of 50 to 90%. The response time is 3.3 s, while the recovery time is 3.6 s. A syringe pump was employed to introduce air with a specified humidity into the microfluidic chip channel at a specific rate. Visualization of the relative humidity changes in the microfluidic chip channel was undertaken in conjunction with COMSOL Multiphysics 6.2 software. The relative humidity in the channel was observed over a designated period of time and compared with the morphology of the spores in the experimental phenomenon. The results verified that the model was accurate, indicating that the relative humidity in the channel at this time could be approximately measured based on the morphology of the spores in the channel. We also exploited a microscale humidity sensor integrated with a smartphone-based microscopy for skin humidity sensing. The difference in spore morphology between gloved and ungloved fingers near the filter membrane of the device in the experiment demonstrates the feasibility of the application in measuring the humidity of finger skin.

The experimental results demonstrate that the microscale humidity sensor exhibits a satisfactory response to changes in relative humidity. However, other potential limitations exist: (1) generally, we can transport the spore through high-speed air flow and with the assistance of a magnetic field, yet transporting the humidity microsensors to some deeper or smaller position of the microchip may be harder to conduct; (2) instead of collecting the electric signals that respond to environmental humidity, we need microscopy or the tailor-made portable smartphone device to observe the status of the sensor under the microenvironment. Future improvements should focus on enabling the capture of a spore morphology image, the measurement of spore length through data analysis, and the direct display of the present humidity value. The prospective expansion of the application of spores with magnetic properties, subsequent to sputtering iron, is anticipated to facilitate novel breakthroughs in practical applications, including the directional movement of spores in conjunction with magnetic fields [39]. Furthermore, there is hope that it will be eliminate the need for a microscope by utilizing sputtered iron to convey an electrical signal in response to ambient humidity.

## Data Availability

The data presented in this study are available upon request from the corresponding author.

## Supplementary Materials

The following supporting information can be downloaded at https://www.mdpi.com/article/10.3390/bios14090414/s1: Figure S1: Device for morphology of spores at different relative humidities; Figure S2: Real images show measurement of the humidity changes in spores in microfluidic chip channels; Figure S3: (A) Plots of spore’s extent corresponding to different humidity, in which sensor 1, 2 and 3 represent three different microsensors before iron sputtering, (B) Plots of spore’s extent corresponding to different humidity, in which sensor 4, 5 and 6 represent three different microsensors after iron sputtering, and (C) show corresponding normalized humidity card for sensor 1, 2 and 3, (D) show corresponding normalized humidity card for sensor 4, 5 and 6; Figure S4: Time lapse images showing status of the humidity microsensor in the channel of the microchip when a dry air with 1 mm/s was injected; Figure S5: Two different fragments of the microsensor to work in the space smaller than 400 μm. Please note that these images are taken from Figure 4B; Figure S6: (A) The stretching or curling of microsensor is restricted near the corner, (B) Change the humidity from high to low and low to high repeatedly to make the slight movement of the microsensor, (C) The microsensor is get rid from the restriction; Figure S7: Long-term stability of the iron sputtered spores, here the same batch of spores were sputtered with iron and measured both within one week and after 57 days; Figure S8: (A) morphology of spores following exposure to 100 °C for up to 30 minutes, (B) morphology of spores under varying relative humidity conditions; Table S1: Comparison of the response/recovery time with other sensors previously reported; Table S2: Comparison of sensitivity and accuracy with other sensors previously reported; Video S1: Microscale humidity sensor combined with smartphone-based microscopy for skin humidity measurement [40,41,42,43,44,45,46,47].
