# Supplementary material for: Microscale Humidity Sensor Based on Iron-Coated Elaters of Equisetum Spores
Source: Biosensors (Basel). 2024 Aug 26;14(9):414. doi: 10.3390/bios14090414 (PMC11430200; doi:10.3390/bios14090414)
Supplement: Supplementary file 1 [file biosensors-14-00414-s001.zip › biosensors-3145741-supplementary.pdf]

biosensors

Supplementary Materials

Microscale Humidity Sensor Based on Iron-Coated Elaters of *Equisetum* Spores

Yanting Liu [1], Zhexuan Lin [1], Xiaochun Li [2], Rui Huang [1], Xuewan Wu [1], Ruyi Deng [1] and Kaisong Yuan [1,*]

1 Bio-Analytical Laboratory, Shantou University Medical College, Shantou 515041, China; 23ytliu1@stu.edu.cn (Y.L.); g_zxlin@stu.edu.cn (Z.L.); 22rhuang1@stu.edu.cn (R.H.); 23xwwu@stu.edu.cn (X.W.); 20rydeng@stu.edu.cn (R.D.)

2 Department of Ultrasound, First Affiliated Hospital of Shantou University Medical College, Shantou 515041, China; 23xcli@stu.edu.cn

* Correspondence: ksyuan@stu.edu.cn

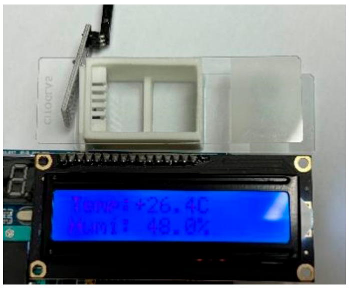

Figure S1. Device for morphology of spores at different relative humidities.

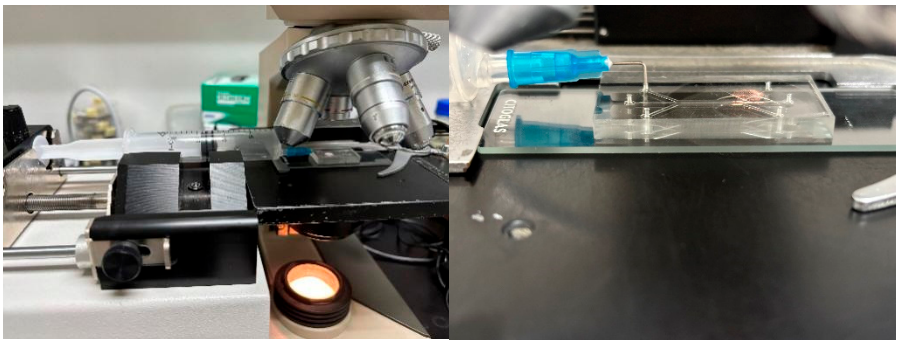

Figure S2. Real images show measuring the humidity changes of spores in microfluidic chip channels.

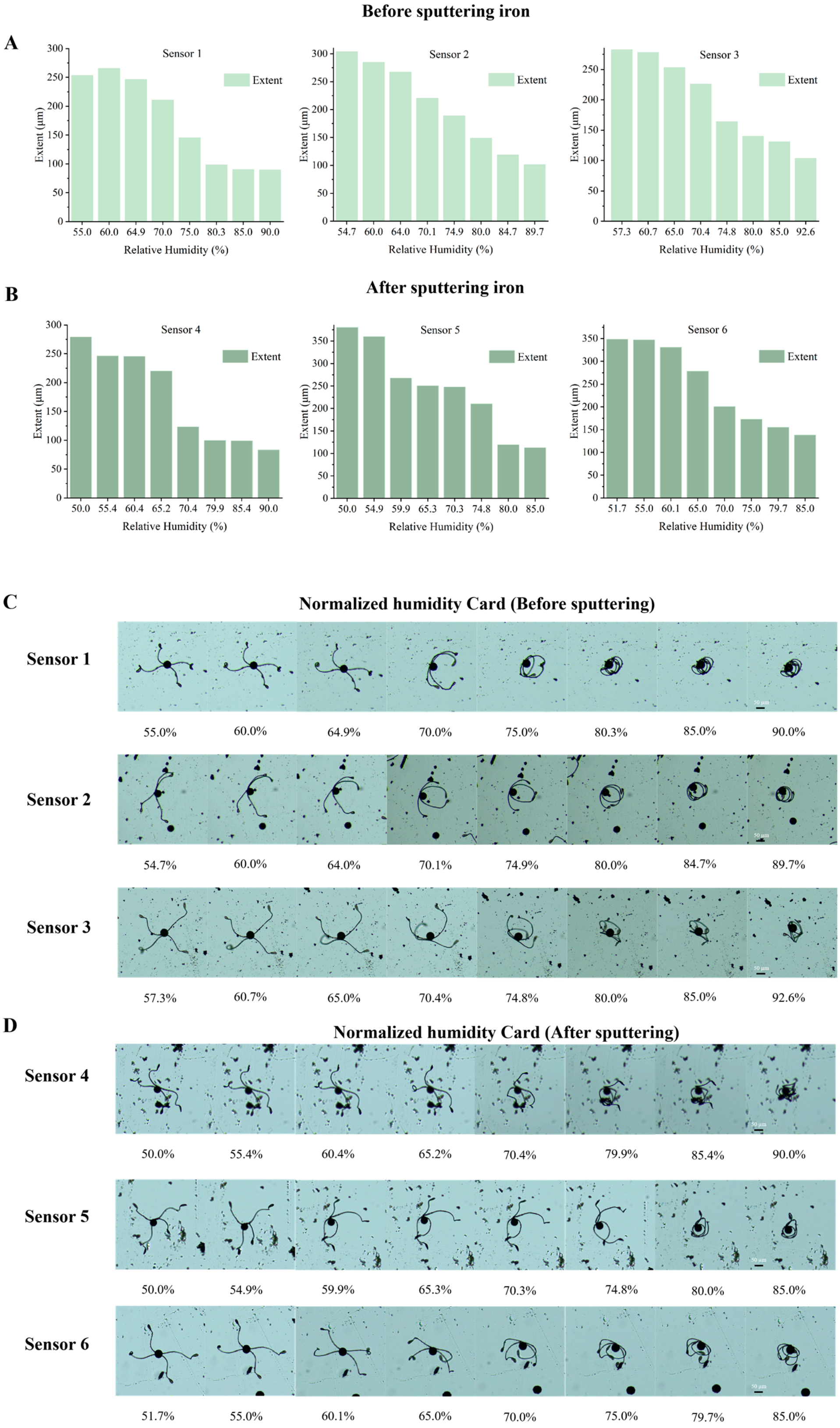

Figure S3. (A) Plots of spore's extent corresponding to different humidity, in which sensor 1, 2 and 3 represent three different microsensors before iron sputtering, **(B)** Plots of spore's extent corresponding to different humidity, in which sensor 4, 5 and 6 represent three different microsensors after iron sputtering, and **(C)** show corresponding normalized humidity card for sensor 1, 2 and 3, **(D)** show corresponding normalized humidity card for sensor 4, 5 and 6.

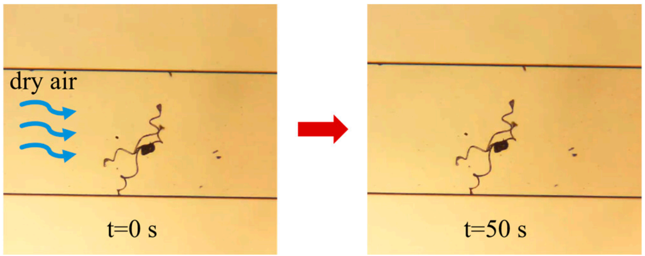

Figure S4. Time lapse images showing status of the humidity microsensor in the channel of the microchip when a dry air with 1 mm/s was injected. Please note that these pictures are taken from the measurement described in Figure 4A, the time when dry air was injected.

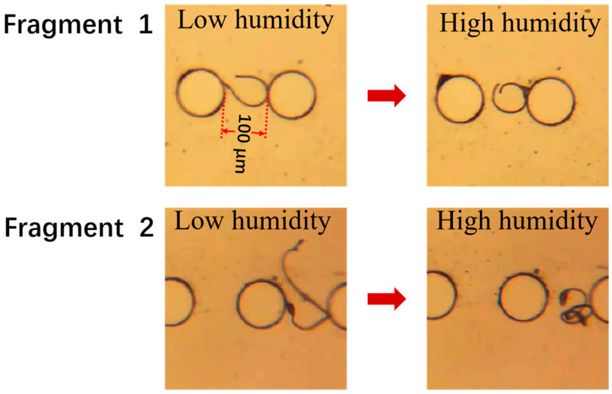

Figure S5. Two different fragments of the microsensor to work in the space smaller than 400 μm. Please note that these images are taken from Figure 4B.

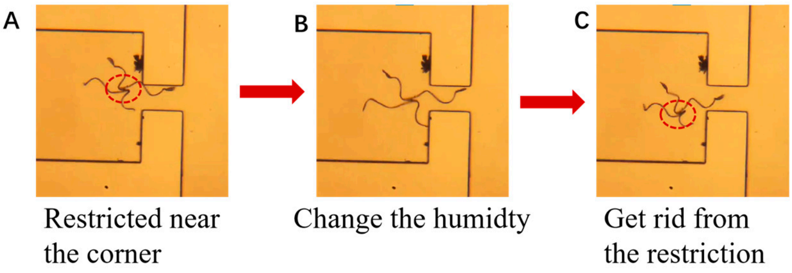

Figure S6. **(A)** The stretching or curling of microsensor is restricted near the corner, **(B)** Change the humidity from low to high and high to low repeatedly to make the slight movement of the microsensor, **(C)** The microsensor is get rid from the restriction.

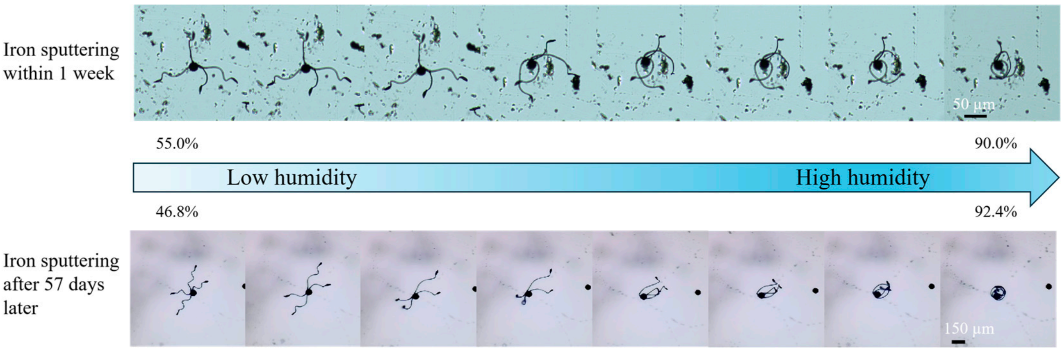

Figure S7. Long-term stability of the iron sputtered spores, here the same batch of spores were sputtered with iron and measured both within one week and after 57 days. (please note that for the sample "sputtering within 1 week", we measure it using the same spore as in Figure S3D, sensor 5).

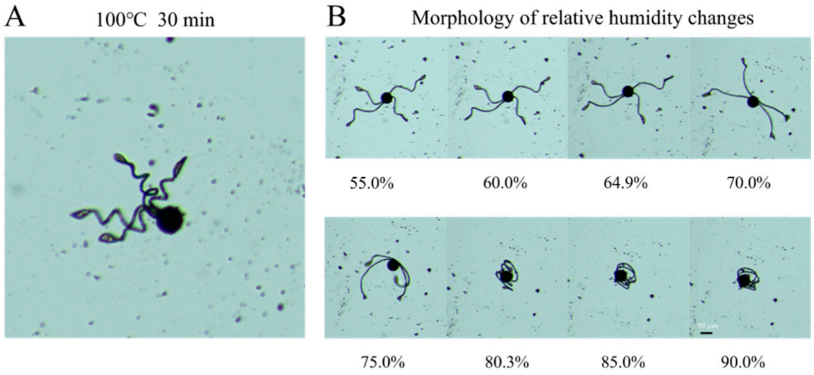

Figure S8. (A) morphology of spores following exposure to 100°C for up to 30 minutes, **(B)** morphology of spores under varying relative humidity conditions.

Table S1. Comparison of the response/recovery time with other sensors previously reported.

Materials	Response time	Recovery time	Ref
Suspended functionalized carbon nanotubes	12s	47s	1
Indium hydroxide ($In(OH)_3$) nanoparticles	14s	204s	2
Fe^{3+} ions doped with SnO_2 (Fe/SnO_2)	10s	8s	3
Porous SnO_2/TiO_2 composite ceramics modified with Mo and Zn	18s	27s	4
Iron-coated elaters of *Equisetum* spores	3.3s	3.6s	This work

Table S2. Comparison of sensitivity and accuracy with other sensors previously reported.

Materials	Sensitivity	Accuracy	Ref
Hollow MoS_2 micro@nano-spheres	32.19 nF/% RH	N.A.	5
AgNFs/MWCNT/SA	N.A.	Sensitive to the humidity difference of 5% in the high humidity limit	6
Quartz crystal microbalance	262.21 Hz/%RH	N.A.	7
Vertically Aligned ZnO Nanorods and Graphene Oxide	196.95%	±0.37pF	8
Iron-coated elaters of *Equisetum* spores	5.4 µm/%RH	About 5 %	This work

References

1. Arunachalam, S.; Izquierdo, R.; Nabki, F. Low-hysteresis and fast response time humidity sensors using sus-pended functionalized carbon nanotubes. *Sensors* **2019**, *19*, 680.
2. Zhao, Z.; Meng, X.; Pan, Y.; Jin, G.; Shen, X.; Wu, L. Humidity Sensor Based on $In(OH)_3$ Nanoparticles. *ACS Appl. Nano Mater.* **2024**, *7*, 16498–16505. https://doi.org/10.1021/acsanm.4c02529.
3. Zhang, H.; Zhang, H.; Man, J.; Chen, C. Preparation of high performance Fe-doped SnO2 humidity sensor and its application in respiration detection. *Sens. Actuators A Phys.* **2023**, *362*, 114644.
4. Sekulić, D.L.; Ivetić, T.B. Characterization of an Impedance-Type Humidity Sensor Based on Porous SnO2/TiO2 Composite Ceramics Modified with Molybdenum and Zinc. *Sensors* **2023**, *23*, 8261.
5. Tan, Y.; Yu, K.; Yang, T.; Zhang, Q.; Cong, W.; Yin, H.; Zhang, Z.; Chen, Y.; Zhu, Z. The combinations of hollow MoS_2 micro@ nano-spheres: one-step synthesis, excellent photocatalytic and humidity sensing properties. *J. Mater. Chem. C* **2014**, *2*, 5422–5430.

6. Liu, L.; Tan, H.; Zhang, L.; Huang, Y.; Xiang, C.; Li, M.; Wang, W.; Wang, D. Flexible Humidity Sensing Fiber with High Sensitivity and Stability for Wearable Weaving and Physiological Signal Monitoring. *ACS Appl. Nano Mater.* **2024**, *7*, 14458–14467. https://doi.org/10.1021/acsanm.4c01940.
7. Chen, Q.; Yao, Y.; Huang, X.-h.; Liu, D.; Mao, K.-l. Simulation analysis and experimental verification for sensitivity of IDE-QCM humidity sensors. *Sens. Actuators B Chem.* **2021**, *341*, 129992.
8. Pongampai, S.; Pengpad, P.; Meananeatra, R.; Chaisriratanakul, W.; Poyai, A.; Horprathum, M.; Chananonnawathorn, C.; Titiroongruang, W.; Muanghlua, R. Sensing layer combination of vertically aligned ZnO nanorods and graphene Engineering. *IEEJ Trans. Electr. Electron. Eng.* **2020**, *15*, 965–975. https://doi.org/10.1002/tee.23140.
